# Influence of survival, promotion, and growth on pattern formation in zebrafish skin

**DOI:** 10.1038/s41598-021-89116-4

**Published:** 2021-05-10

**Authors:** Christopher Konow, Ziyao Li, Samantha Shepherd, Domenico Bullara, Irving R. Epstein

**Affiliations:** 1 Department of Chemistry, Brandeis University, Waltham, MA 02453 USA; 2 Department of Chemistry and Biochemistry, University of Oregon, Eugene, OR 97403 USA

**Keywords:** Dynamical systems, Nonlinear dynamics, Numerical simulations, Stochastic modelling

## Abstract

The coloring of zebrafish skin is often used as a model system to study biological pattern formation. However, the small number and lack of movement of chromatophores defies traditional Turing-type pattern generating mechanisms. Recent models invoke discrete short-range competition and long-range promotion between different pigment cells as an alternative to a reaction-diffusion scheme. In this work, we propose a lattice-based “Survival model,” which is inspired by recent experimental findings on the nature of long-range chromatophore interactions. The Survival model produces stationary patterns with diffuse stripes and undergoes a Turing instability. We also examine the effect that domain growth, ubiquitous in biological systems, has on the patterns in both the Survival model and an earlier “Promotion” model. In both cases, domain growth alone is capable of orienting Turing patterns above a threshold wavelength and can reorient the stripes in ablated cells, though the wavelength for which the patterns orient is much larger for the Survival model. While the Survival model is a simplified representation of the multifaceted interactions between pigment cells, it reveals complex organizational behavior and may help to guide future studies.

## Introduction

Morphogenesis, the symmetry-breaking phenomenon in which a uniform mass (such as an embryo) spontaneously develops complex yet organized heterogeneities in a reproducible manner^1,2^, has fascinated biologists and mathematicians for decades. One influential theory of morphogenesis, developed by Alan Turing^1^, explains how a simple two-morphogen reaction-diffusion system can spontaneously develop spatially periodic, temporally stationary structures. Gierer and Meinhardt later rederived and expanded on this idea, emphasizing the concept of short-range activation and long-range inhibition (SRALRI), whereby an activator is able to locally increase its concentration (usually in an autocatalytic manner), and an inhibitor is able to diffuse faster (over a longer range) and inhibit the activator^3–5^. However, many biologists shied away from Turing’s theory due to the lack of concrete experimental evidence^6,7^, Turing’s original physically unrealistic models^1,5^, and extreme sensitivity to reaction parameters^2,7–9^. Even when chemists in the early 1990s (almost 40 years after Turing’s original work was published) provided experimental evidence of Turing-type patterns in an inorganic reaction-diffusion system^6,10^, few biologists took note.

This situation began to change when researchers noticed that the skin patterning on various types of fish bore striking resemblance to patterns simulated with Turing’s mechanism (commonly called Turing patterns)^11^, both in their morphology and in the manner in which the patterns develop as the fish matures. This led to a boom in Turing pattern-related research, and many examples of biological patterning were modeled with Turing-type interactions^12–15^. Zebrafish (*Danio rerio*) quickly emerged as a model system for biological patterning studies, as the fish grow quickly and their genome has been fully sequenced^16,17^. In addition, their semi-translucent skin allows for imaging of the chromatophores, the colored cells that make up the skin patterns, with basic low-powered microscopes^14,15,17^. These studies have shown that there are three major types of chromatophores that play a role in pattern formation: black melanophores, yellow xanthophores, and light blue/silvery iridophores^15,17–19^. As the zebrafish develops past its larval stage, xanthophores form on the skin, guiding the differentiation of stem cells into iridophores, which are attracted to the xanthophores, and melanophores, which are repelled. This leads to the formation of an initial pre-pattern arrangement of periodic black melanophore stripes and yellow xanthophore interstripes along the body and fins of the zebrafish, which guides the future orientation of the fully developed pattern^15,18,20^.

Chromatophores are not traditional “morphogens”, at least in terms of Turing’s original theory^1^. They do not “react” in a chemical manner, but rather interact with each other at different length scales^15,19–22^. Empirical studies indicate that adjacent melanophores and xanthophores inhibit each other^15,20^, and that xanthophores may stimulate the production and survival of remote melanophores *via* a Delta-Notch signaling pathway^15,19,21,22^. Melanophores extend long projections towards xanthophores. These projections carry a signal from transmembrane *deltaC* and *delta-like 4* proteins on the xanthophores to *notch1a* and *notch2* receptors on the melanophores^22^. The projections grow to a maximum length of half the distance between two adjacent stripes. This signaling is critical for melanophore survival and growth, as ablation of xanthophores in an interstripe (i.e., a xanthophore-dense stripe) leads to a decrease in the density of melanophores in neighboring stripes^15,23^. Although these interactions are not “reactions” in a typical chemical sense, their combined effect produces dynamical effects in agreement with Gierer and Meinhardt’s SRALRI criterion for Turing patterns^3,17^.

The lack of significant cell movement in zebrafish is difficult to reconcile with Turing’s theory, which is based on reaction and diffusion of an inhibitor. Some studies have considered mixtures of diffusing and non-diffusing morphogens, but only in some cases does this lead to robust Turing-type patterns^8,9,24^. While the zebrafish cells do move around somewhat^18–20,25,26^, only iridophores regularly show large amounts of movement along the skin. Even so, it is unclear if iridophores are essential to pattern formation, as they are not present on the fins of the zebrafish, which are patterned in a similar form to the body (see^18,25,27^ for an active debate on this topic, and^19,28^ for more in-depth reviews of the two positions). Xanthophores and melanophores also move slightly in a “run and chase” mode, in which a small dendrite in the xanthophore causes it to follow the faster-moving melanophore^20,26^. However, this amount of movement is not sufficient to serve as the sole driver of pattern formation, as the diffusion length of the cellular motion does not correlate with the wavelength of the pattern^26^. Thus, the origin of these patterns is not a diffusively-driven instability in the sense of a traditional Turing pattern-forming system. All of this behavior, both movement and cellular interactions, is captured and analyzed *in silico* in the agent-based model developed by Volkening and Sandstede^29,30^.

**Figure 1:**
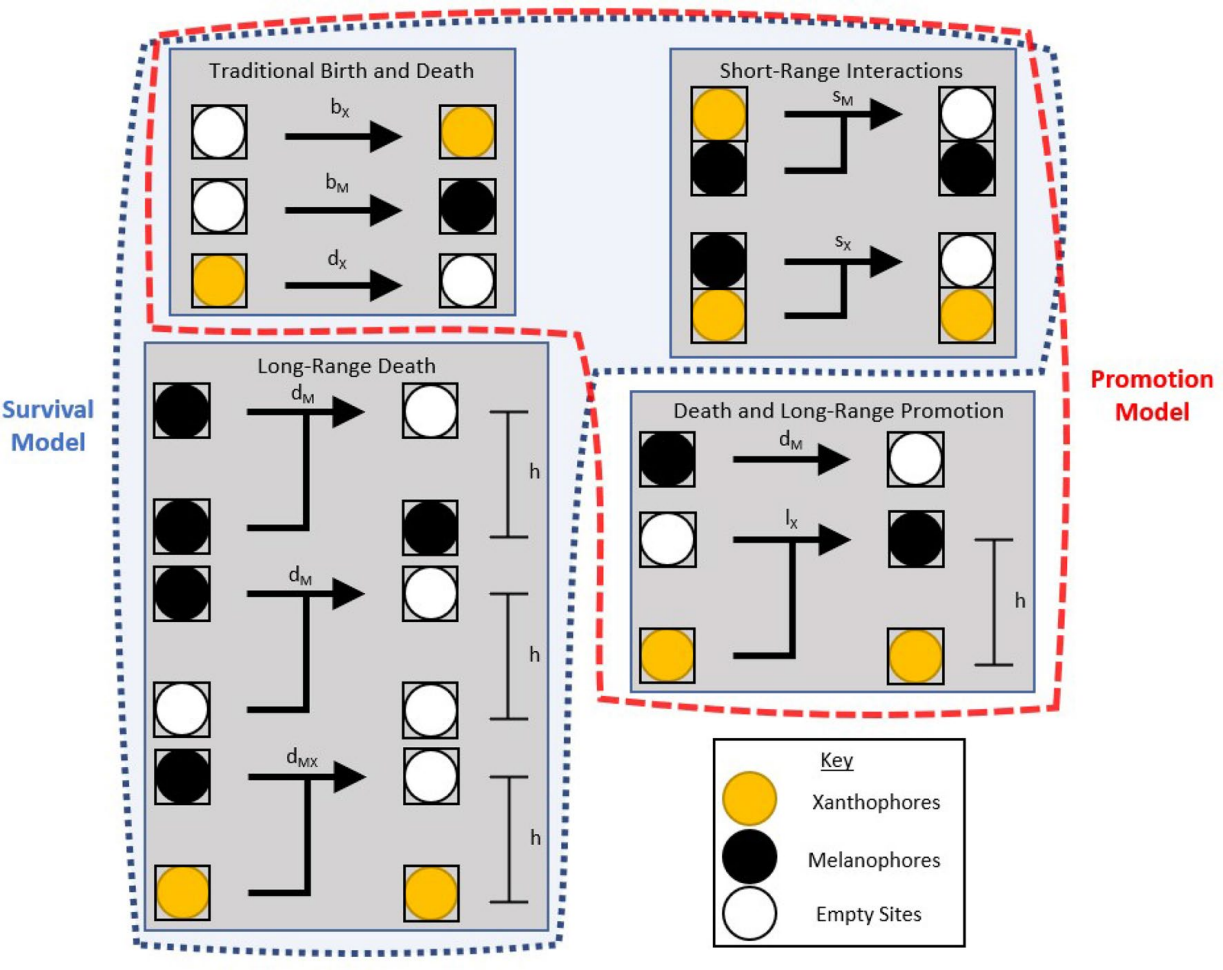
Schematic depicting the interactions of chromatophores in both the Promotion model and the Survival model of zebrafish skin pattern formation. The blue outline shows the Survival model, and the red outline shows the Promotion model.

While this agent-based formulation captures a significant amount of biological detail, Bullara and De Decker took a more conceptual modeling approach^31^. They modeled the fish skin as a two-dimensional lattice, with each lattice site either empty or occupied by a melanophore or xanthophore. Melanophores and xanthophores inhibited each other when adjacent, and xanthophores promoted melanophore birth a distance *h* away^31^. A schematic of these interactions can be seen outlined in red in Fig. 1. Since the long-range feedback responsible for the pattern generation in this model is xanthophores promoting the growth of new melanophores, we refer to this model as the “Promotion model”^31^.

In the present work, we propose a lattice-based “Survival model” which is inspired by experimental evidence as to the nature of the long-range interactions between the chromatophores^21,22^. In this model,we suggest that the xanthophores increase the survival rate of melanophores indirectly at a distance *h*, as shown in Fig. 1 (outlined in blue). We examine both analytically and numerically the ability of this new model to generate Turing patterns without diffusion. Our studies indicate that the patterns that emerge from the Survival model arise from a Turing instability, and provide additional evidence that zebrafish patterning may be the result of a Turing-type mechanism.

We then examine the impact of *domain growth* on pattern formation in both the Survival and Promotion models. Domain growth has been an emergent area of interest in studies of morphogenesis from mathematical^32–35^, chemical^36,37^, and biological^11,23,30,38^ standpoints. In particular, studying the effect of domain growth facilitates comparison to the results of other models, including continuous-field approaches^38^ as well as agent-based models^29,30^. We show that domain growth alone is sufficient to orient the simulated patterns along the growing axis for both the Survival and Promotion models. However, the patterns orient at significantly different long-range interaction distances.

## Results

### The Survival model on a lattice

We first propose the Survival model, which includes a novel form of the long-range interaction between chromatophores, as seen outlined in blue in Fig. 1. The conceptual idea behind the Survival model is to separate the death of a melanophore into three separate processes, where a different species occupies one or more nodes a distance *h* away from the reference melanophore. When this species is a xanthophore, we assume that the melanophore death rate is significantly less than if another melanophore or no chromatophores occupies this space. This form of long-range interaction is inspired by recent research from Hamada et al., which indicates that remote xanthophores enhance the survival of melanophores^22^.

To implement the above idea, we model the skin of a zebrafish as a lattice with discrete, non-diffusive interactions between nodes (cells). Each node can be occupied by a yellow xanthophore (*X*), a black melanophore (*M*), or an empty site (*S*, depicted in white in all figures). This approach contrasts with most other studies of biological pattern formation, which rely on continuous variables that undergo diffusion-like motion and are modeled with partial differential equations (PDEs)^11,14,17,38^. We choose to use the discrete lattice-based modeling approach because most of the interactions between the chromatophores can be classified as either short-range^15,39^ or long-range interactions^19,21,22^ that do not require significant cell movement. For a full discussion of the rationale for this approach to modeling, see Bullara and De Decker^31^. The long-range interactions in the Survival model are represented as:

$$\begin{aligned}&M_{i} + M_{i \pm h} {\mathop {\longrightarrow }\limits ^{[d_M]}} S_{i} + M_{i \pm h}\\&M_{i} + S_{i \pm h} {\mathop {\longrightarrow }\limits ^{[d_M]}} S_{i} + S_{i \pm h}\\&M_{i} + X_{i \pm h} {\mathop {\longrightarrow }\limits ^{[d_{M X}]}} S_{i} + X_{i \pm h} \end{aligned}$$

where *i* is the lattice node (explicitly shown on a one-dimensional lattice in the equations), and *h* is the distance of the long-range interaction (usually the width of one stripe^22^). The melanophore dies at a rate $d_M$. However, if a xanthophore occupies a node at a distance *h* away from the melanophore, we set the death rate to $d_{M X} \ll d_M$ in order to portray the enhancement of melanophore survival resulting from the presence of distant xanthophores. As shown in Fig. 1, these reactions take the place of the simple death of melanophores. There is no long-range promotion of melanophore birth in the Survival model.

In addition to the new long-range interactions, the Survival model includes short-range competition reactions between melanophores and xanthophores given by:

$$\begin{aligned}&X_{i} + M_{i \pm 1} {\mathop {\longrightarrow }\limits ^{[s_M]}} S_{i} + M_{i \pm 1}\\&M_{i} + X_{i \pm 1} {\mathop {\longrightarrow }\limits ^{[s_X]}} S_{i} + X_{i \pm 1} \end{aligned}$$

These reactions can be considered self-promoting (or activating), as each chromatophore selectively kills the chromatophore of the opposite type at close range, which then allows for more of itself to be born (and survive) at the resulting unoccupied node. The Survival model also includes simple birth of both melanophores and xanthophores,

$$\begin{aligned}&S_i {\mathop {\longrightarrow }\limits ^{[b_X]}} X_{i}\\&S_i {\mathop {\longrightarrow }\limits ^{[b_M]}} M_{i} \end{aligned}$$

and the simple death of xanthophores.

$$\begin{aligned} X_i {\mathop {\longrightarrow }\limits ^{[d_X]}} S_{i} \end{aligned}$$

Note that - as mentioned above - simple death of melanophores is absent in this model, as it has been replaced by the long-range death processes. A schematic view of all of these reactions can be seen in Fig. 1, where the full Survival model is enclosed by the blue dotted line with the light blue interior.

### Deriving mean field and continuous mean field equations

To investigate the dynamics of the Survival model, we use both stochastic Monte Carlo simulations and deterministic evolution equations. For the evolution equations, we consider the ensemble averages of the Boolean variables $X_i$, $M_i$, and $S_i$ at each lattice node *i*, which are given by $\langle X_i \rangle$, $\langle M_i \rangle$, and $\langle S_i \rangle$ respectively. Using mass-action laws to derive the master equation for this system, and assuming there is no statistical correlation across space (commonly called the mean field assumption, where $\langle A_i,B_j \rangle \simeq \langle A_i \rangle \langle B_j \rangle$), we obtain the following system of ordinary differential equations:

1 $$\begin{aligned}&\frac{d \langle X_i \rangle }{d t} = b_X \langle S_i \rangle - d_X \langle X_i \rangle - \frac{1}{2} s_M \langle X_i \rangle \left( \langle M_{i+1} \rangle + \langle M_{i-1} \rangle \right) \end{aligned}$$

2 $$\begin{aligned}\frac{d \langle M_i \rangle }{d t} &= b_M \langle S_i \rangle - \frac{1}{2} s_X \langle M_i \rangle \left( \langle X_{i+1} \rangle + \langle X_{i-1} \rangle \right) - \frac{1}{2} d_{M X} \langle M_i \rangle \left( \langle X_{i+h} \rangle + \langle X_{i-h} \rangle \right) \nonumber \\&\quad- \frac{1}{2} d_M \langle M_i \rangle \left( \langle S_{i+h} \rangle + \langle S_{i-h} \rangle + \langle M_{i+h} \rangle + \langle M_{i-h} \rangle \right) \end{aligned}$$

Note that we do not require a separate equation for $\langle S_i \rangle$ because the three variables at site *i *are related by the balance $\langle S_i \rangle = 1 - \langle X_i \rangle - \langle M_i \rangle$.

We transform Eqs. (1)–(2) into a system of partial differential equations (PDEs) by switching to a continuous spatial coordinate $r = i a$, where *a* is the average diameter of a cell, on which we define the continuous field variables $x = x(r) = \langle X_i \rangle$ and $m = m(r) = \langle M_i \rangle$, which are assumed to change smoothly over *r*. Using a second-order Taylor series expansion in *r*, we obtain the following continuous mean field PDE system:

3 $$\begin{aligned}&\frac{\partial x}{\partial t} = b_{x} (1-x-m) - d_{x} x - s_{M} m x - \frac{a^2}{2} s_{M} x \nabla ^2 m \end{aligned}$$

4 $$\begin{aligned}&\frac{\partial m}{\partial t} = b_{M} (1-x-m) - d_M m - ({-}d_M + d_{M X} + s_X) x m - \left( \frac{a^2}{2} s_X m + \frac{h^2 a^2}{2} d_{M X} m - \frac{h^2 a^2}{2} d_M m \right) \nabla ^2 x \end{aligned}$$

It is important to remember that the apparent cross-diffusion terms in Eqs. (3)–(4) do not represent true diffusion, but rather are a result of the continuous approximation of the short-range and long-range *discrete* interactions. While Eqs. (1)–(4) describe a one-dimensional lattice (Eqs. (1)–(2)) or one-dimensional coordinate system (Eqs. (3)–(4)), the same derivation can be used to extend the system to higher dimensions. For the theoretical basis for the lattice approach to modeling and the equations, see the Methods section.

### Spatial patterns arising from the Survival model are Turing-type patterns

We use a variety of methods to study the Survival model and show how it can generate Turing patterns. In the simulations presented here, we limit ourselves to an idealized case where we assume that the short-range mutual inhibition rate constants are identical ($s_X = s_M = s$) and that the death of xanthophores is caused only by short-range and long-range interactions, and not by simple decay ($d_X = 0$). In addition, we assume that the “survival” signal given to the melanophores by the xanthophores effectively prevents the melanophores from dying ($d_{M X} = 0$). While biologically unrealistic, these approximations allow us to qualitatively represent the dynamic behavior of the system, as well as allow a closer comparison to the previously published results of the Promotion model^31^, which were obtained under similar assumptions.

To begin, we perform a linear stability analysis (LSA) on the continuous mean field PDE system (Eqs. (3)–(4)) under the conditions of our simulations ($d_X = d_{M X} = 0$, $s_X = s_M = s$). A detailed description of the LSA is included in the the Methods section. The system admits two homogeneous steady states:

5 $$\begin{aligned}&x_1 = 1, \quad m_1 = 0 \end{aligned}$$

6 $$\begin{aligned}&x_2 = \frac{b_X d_M}{b_X (d_M - s) + b_M s}, \quad m_2 = \frac{(b_M - b_X) s}{b_X (d_M - s) + (b_M + d_M) s} \, . \end{aligned}$$

The LSA shows that when the parameter *h* is larger than a critical value $h_T$ (which is controlled by the values of the other parameters), the second steady state can undergo a Turing bifurcation, which is generally associated with spontaneous generation of stationary patterns like those observed in the MC simulations. The Turing bifurcation arises in response to a perturbation of wavenumber $k_T$, and at the bifurcation point would generate a pattern with the critical wavelength $\lambda _T = 2 \pi / k_T$. For more details of these calculations, see the Methods section.

**Figure 2:**
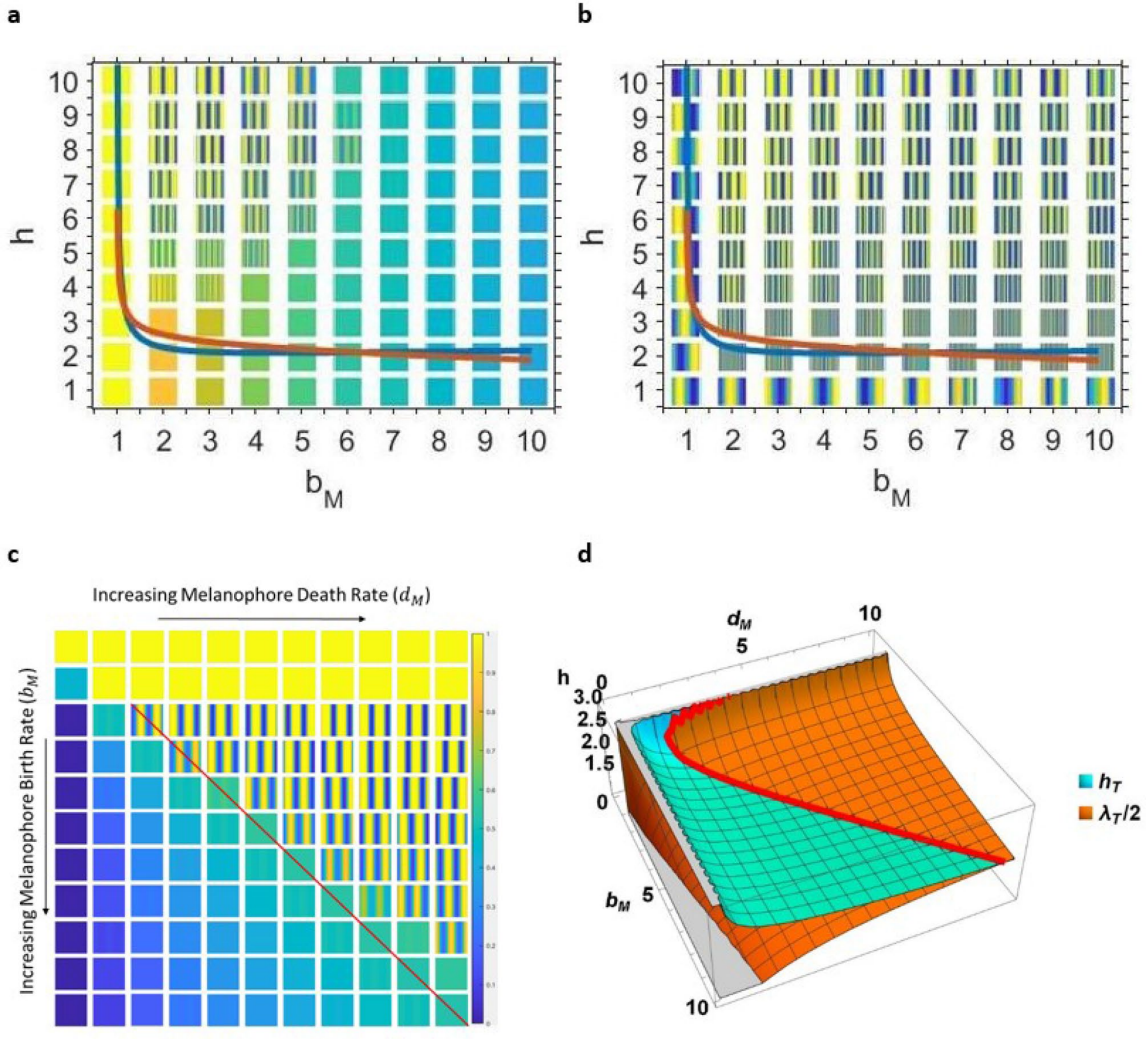
Numerical simulations and results of LSA of the Survival model in one dimension. For each numerical simulation, Eqs. (1) and (2) were simulated on a size $n=50$ lattice with periodic boundaries. The xanthophore concentration ($\langle X_i \rangle$) is shown. The results were extended vertically into a square shape for ease of viewing. The following conditions were held constant in all simulations and when performing the LSA: $b_X = s = 1$, $d_X = d_{M X} = 0$. (**a**) and (**b**) Simulations of the Survival model for various long-range interaction distances *h* and melanophore birth rates $b_M$. The death rate of melanophores was held constant at $d_M = 4$. The blue curve is a plot of the minimum long-range interaction distance $h_T$ that allows for Turing patterns, and the orange curve is one-half of the critical wavelength ($\lambda _T / 2$). The simulations in (**a**) and (**b**) are identical, but (**a**) is absolutely scaled between a normalized concentration of zero and one, while (**b**) is relatively scaled between the minimum and maximum values of $\langle X_i \rangle$ for that simulation. (**c**) Simulations of the Survival model for various $b_M$ and $d_M$ parameter combinations. For all simulations, the long-range interaction distance was held constant at $h = 15$. The red line approximately indicates the onset of patterning at $h_T = \lambda _T /2$. (**d**) Analytical result of LSA of the continuous mean field Eqs. (3)–(4). The cyan surface is a plot of the bifurcation value $h_T$, the critical long-range interaction distance. The orange surface is a plot of half the critical wavelength, $\lambda _T / 2$. The red curve shows the intersection of the two surfaces, where $h_T = \lambda _T /2$.

We then compare the results of the LSA to simulations of the one-dimensional mean field equations (Eqs. (1)–(2)) on a lattice of size $n=50$ with periodic boundary conditions, as shown in Fig. 2. The simulations are extended vertically for ease of viewing and show the normalized concentration of xanthophores at each lattice node ($\langle X_i \rangle$). The blue curves in Fig. 2a,b show the bifurcation parameter $h_T$, and the orange curve shows half the critical wavelength $\lambda _T / 2$. Based on the LSA, we would expect to see Turing patterns everywhere above the blue $h_T$ curve. However, when we examine the absolutely scaled (between 0 and 1) mean field simulations, we see that large amplitude patterns only form for part of the parameter space (Fig. 2a). It is only when we examine the relatively scaled (between the minimum and maximum $\langle X_i \rangle$ values for that simulation) simulations that Turing patterns can be distinctly seen for all the parameter values in the region past the Turing bifurcation point (Fig. 2b). So although the LSA correctly predicts the region of the parameter space where patterns emerge, it cannot account for the fact that some of these patterns have an extremely small amplitude.

We also explore the relationship between multiple Survival model “reaction” parameters. In Fig. 2c, we show simulations of the mean field equations (Eqs. (1)–(2)) at a constant long-range interaction distance $h = 15$ as we vary the birth and death rates of melanophores ($b_M$ and $d_M$ respectively). We observe that large-amplitude patterns form only when $d_M > b_M s$ (we have marked the $d_M = b_M s$ line in red in Fig. 2c). Below this (when $b_M s > d_M$), the patterns have a much smaller amplitude and are not visible in the absolutely scaled images. Interestingly, this line, beyond which patterns can form, also corresponds to the curve $h_T = {\lambda _T /2}$ when the surfaces of $h_T$ (cyan) and $\lambda _T / 2$ are plotted for these $b_M$ and $d_M$ ranges (Fig. 2d). The correspondence between the sharp transition line from small-amplitude to large-amplitude patterns and the curve where $h_T = {\lambda _T / 2}$ persists when parameters $b_X$ and *s* are varied (in Fig. 2, they were fixed at 1). Currently, we are investigating the origin of this sharp increase in pattern amplitude - potentially as a spatial analog in our multiple length scale system of the temporal canard explosion found in some temporally oscillating systems with multiple time scales^40–42^.

### The Survival model produces Turing patterns with disperse melanophore stripes

**Figure 3:**
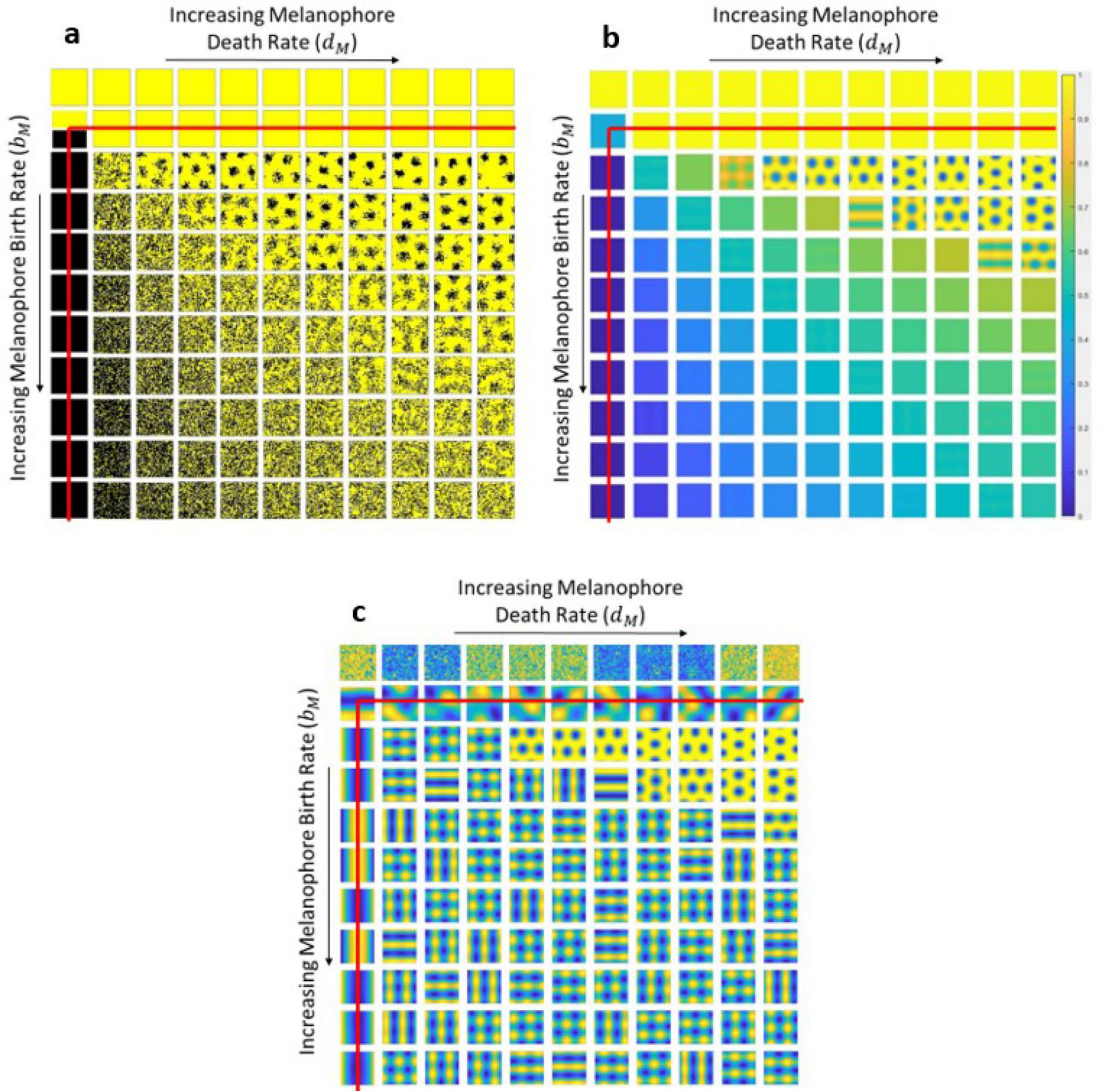
Numerical simulations of the Survival model on a two-dimensional $50 \times 50$ lattice with periodic boundary conditions. In all figures, $b_M$ and $d_M$ were varied from 0 to 10 (top to bottom and left to right, respectively). The following parameters were held constant: $h = 15$, $b_X = s = 1$, $d_X = d_{M X} = 0$. The red lines indicate the area where Turing patterns are predicted by the LSA. (**a**) Stochastic Monte Carlo simulations. Each simulation began on a uniform initial condition corresponding to an empty lattice. Yellow, black, and white lattice sites represent xanthophores, melanophores, and empty sites respectively. (**b**) and (**c**) Numerical integration of the two dimensional mean field equations describing the Survival model. Each simulation started from random initial conditions and ran for 1000 time steps. (**b**) shows the normalized concentration of xanthophores ($\langle X_{i,j} \rangle$) scaled absolutely between zero and one. (**c**) shows the same simulations scaled relative to each simulation’s maximum and minimum.

The one-dimensional mean field simulations and the LSA demonstrate that the patterns resulting from the Survival model are the result of a Turing-type instability. To show the patterns on a two-dimensional domain, we first use a stochastic Monte Carlo method on a static lattice (Fig. 3a). A full description of our simulation algorithm is located in the Methods section, and the code used is available in the Code Availability Section. Figure 3a shows that the relationship between the birth rate of melanophores $b_M$ and their long-range death rate $d_M$ determines whether a pattern is robust enough to be distinguished from the stochastic noise. When we compare these results to deterministic ODE simulations of the two-dimensional mean field equations, we see that the spotted patterns are the only ones that form with a large enough amplitude to be seen with absolute scaling (Fig. 3b) instead of relative scaling (Fig. 3c). However, Turing patterns still exist in the same region of parameter space predicted by the LSA of Eqs. (3)–(4), which is the area below and to the right of the $h_T$ asymptotes (Fig. 2d, cyan curve) marked by the red lines in Fig. 3. When patterns only have small amplitudes, they appear to be overwhelmed by noise in the stochastic simulations, as evidenced in the lower left region of Fig. 3a.

**Figure 4:**
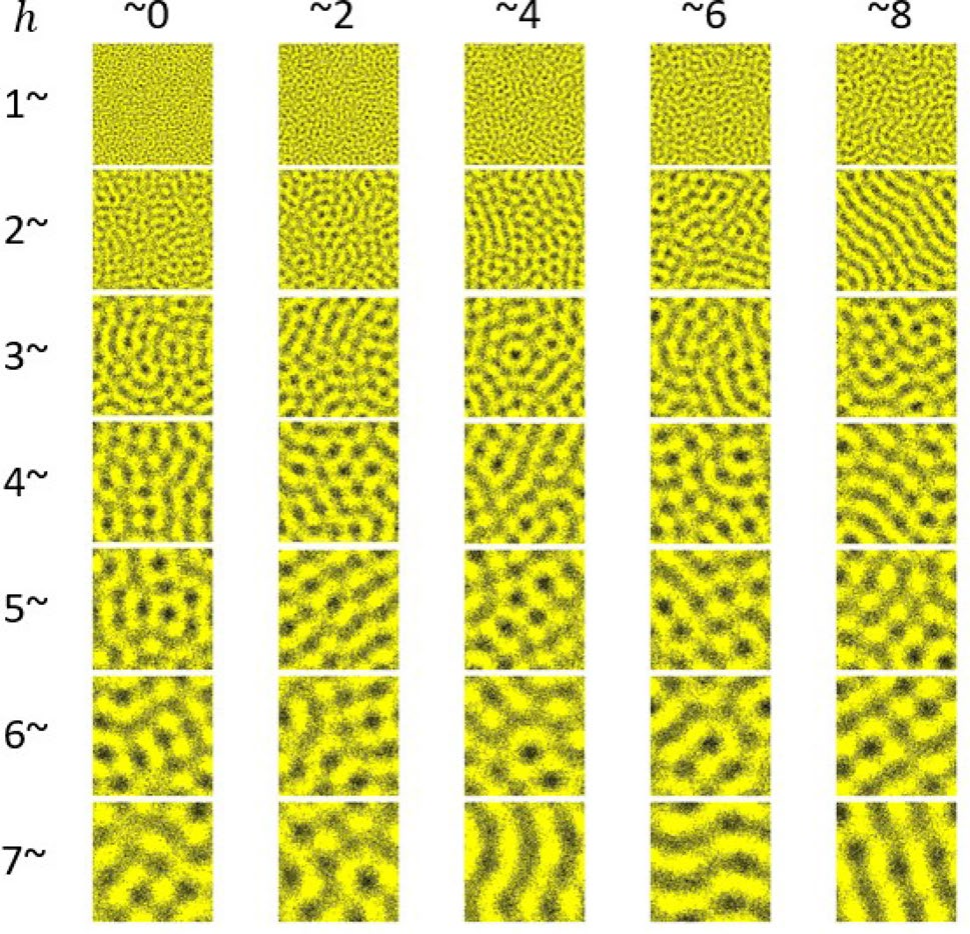
Stationary Turing patterns with varying *h* values in Monte Carlo simulations of the Survival model. Each simulation was performed on a $400 \times 400$ static lattice with periodic boundary conditions. The following parameters were held constant: $b_M = 7$, $d_M = 9$, $b_X = s = 1$, $d_X = d_{M X} = 0$. For each pattern shown, the first digit of the *h* value is given by the row label and the second digit is given by the column label. For example, the Turing pattern in the fifth row and third column has $h = 54$.

Simulations with different *h* values also show that the pattern wavelength - the distance between two stripes of the same color - is roughly given by 2*h*, as seen in Fig. 4. Similar qualitative relationships between stripe distance (pattern wavelength) and the long-range interaction distance found in simulations of the Survival model are also seen in the Promotion model^31^ and agent-based models^29,30^. However, the stochastic simulations of the Survival model show that the areas with melanophores (stripes and spots) are significantly less melanophore-dense than in the Promotion model, with large numbers of xanthophores located in the melanophore stripes (Figs. 3a, 4). In simulations of the Promotion model^31^, melanophore and xanthophore stripes are almost entirely comprised of one chromatophore, with very little intermixing. One possible explanation of this difference is that the survival feedback constitutes a somewhat weaker form of positive feedback than the promotion of new cells. If so, the Survival model may be more strongly affected by the inherent stochastic noise than the Promotion model. We note that that the more “blurred” distribution of cells in the Survival model is closer to that in the actual zebrafish, as loosely-packed xanthophores are found in the stripes of melanophores and more densely-packed xanthophores are isolated in the interstripes^15,19,22,28^.

### Domain growth orients Turing patterns

**Figure 5:**
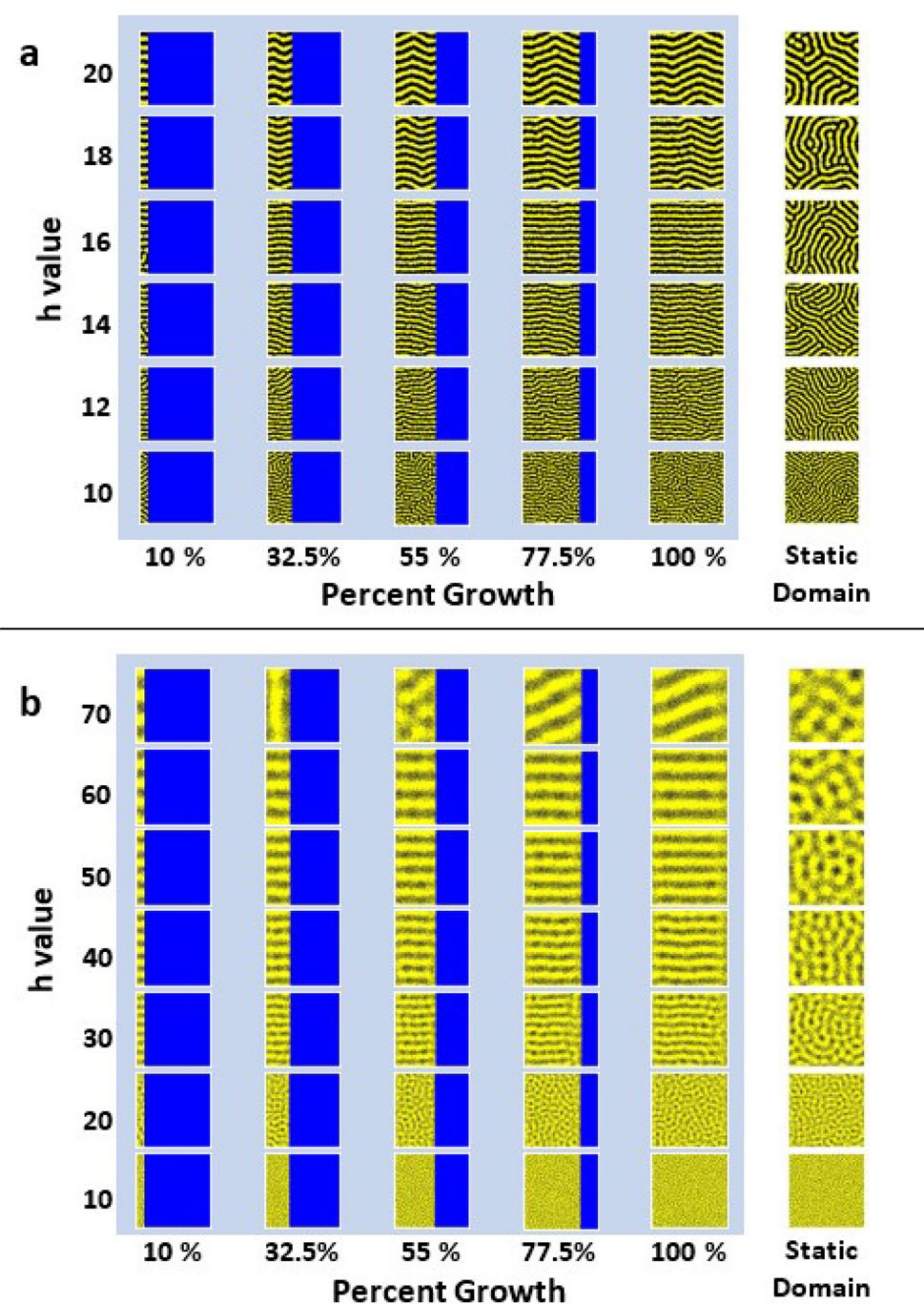
Turing pattern development during domain growth. For each *h* value, two simulations are presented: one on a growing domain (light blue backing) and one on a static domain (right column). For each simulation on a growing domain, images are shown of the developing pattern at 10%, 32.5%, 55%, 77.5%, and 100% of growth. The royal blue areas are the remaining area each simulation will grow into. All simulations are performed with periodic boundary conditions for the same simulation length. (**a**) Stochastic Monte Carlo simulations of the Promotion model on a growing domain. Each simulation begins as a $300 \times 1$ lattice, and grows to a final size of $300 \times 300$. Each simulation is performed with $b_X=1, s=1, l_X=2.5, b_M=d_X=d_M=0$. (**b**) Stochastic Monte Carlo simulations of the Survival model on a growing domain. Each simulation begins as a $400 \times 1$ lattice, and grows to a final size of $400 \times 400$. Each simulation is performed with $b_X = 1, s=1, b_M=7, d_M=9, d_X=d_{M X}=0$.

We examined the impact of domain growth on Turing pattern development in both the Promotion and Survival models (Fig. 5). For this study, we used stochastic Monte Carlo simulations implemented on a lattice, as this yielded morphological behavior similar to deterministic ODE simulations of the mean field equations (Eqs. (1)–(2)) at a fraction of the computing cost. Domain growth was implemented by adding a column of empty lattice sites to the existing lattice after a set number of iterations of the simulation algorithm. See the Methods section for a complete description of the algorithm and the Code Availability section for the code developed for the Survival model simulations. Animations of the the growing simulations for $h = 10$ and $h = 16$ in Fig. 5a (Promotion) and for $h =20$ and $h = 50$ in Fig. 5b (Survival) are contained in the Supplementary Information. Note that for all simulations, we used rate parameters that forced stripe patterns, as it is difficult to distinguish how growth affects spotted patterns, as previously observed in reaction-diffusion systems^37^.

Figure 5 shows that domain growth greatly influences the orientation of the Turing patterns in both the Promotion and Survival models (Fig. 5a,b, respectively). In the Promotion model, for $h \ge 12$, the Turing patterns orient themselves perpendicular to the growing boundary, as shown in Fig. 5a. For larger values of *h*, the stripes become a bit wavy, but overall are still relatively perpendicular to the growing boundary. This is especially evident when compared to the lack of orientation of the static domain simulations under the same conditions (Fig. 5a, right side).

Simulations of the Survival model on a growing domain show results qualitatively similar to the Promotion model. Domain growth still orients the Turing patterns perpendicular to the growing boundary above a specific value of *h* (Fig. 5). However, for the Survival model, the stripes orient themselves in this manner only for $h \ge 30$, a much higher value than in the Promotion model. This is evident in the simulations shown in Fig. 5b, as the resulting Turing patterns for $h = 10$ and 20 show no orientation and look similar to their static domain counterparts.

The pattern orientation behavior for various *h* values occurs regardless of the domain size or shape, as long as the domain is large enough to allow for multiple wavelengths ($\lambda \approx 2 h$). If the domain is unable to contain more than a few wavelengths and its length is not near an integer multiple of a stable wavelength, the stripes may orient obliquely relative to the growing boundary, as seen for the pattern with long-range interaction distance $h = 70$ ($\lambda \approx 140$) for the Survival model in Fig. 5b. If the growth is not one-dimensional (for example, a trapezoid that grows from one side as a model of fin growth, as shown in Supplementary Figure S1) the patterns still orient themselves horizontally with the growing boundary. The stripes added during growth are not affected by any previously formed patterns, as shown in Supplementary Fig. S2. These results show that domain growth significantly affects Turing pattern development and orientation, even in the absence of a pre-pattern. This suggests that growth may play a significant factor in zebrafish skin pattern orientation, especially in the tail and anal fins, which lack iridophores and the horizontal myoseptum^18,23,38,43^.

### Growth can reorient stripes in ablated cells

**Figure 6:**
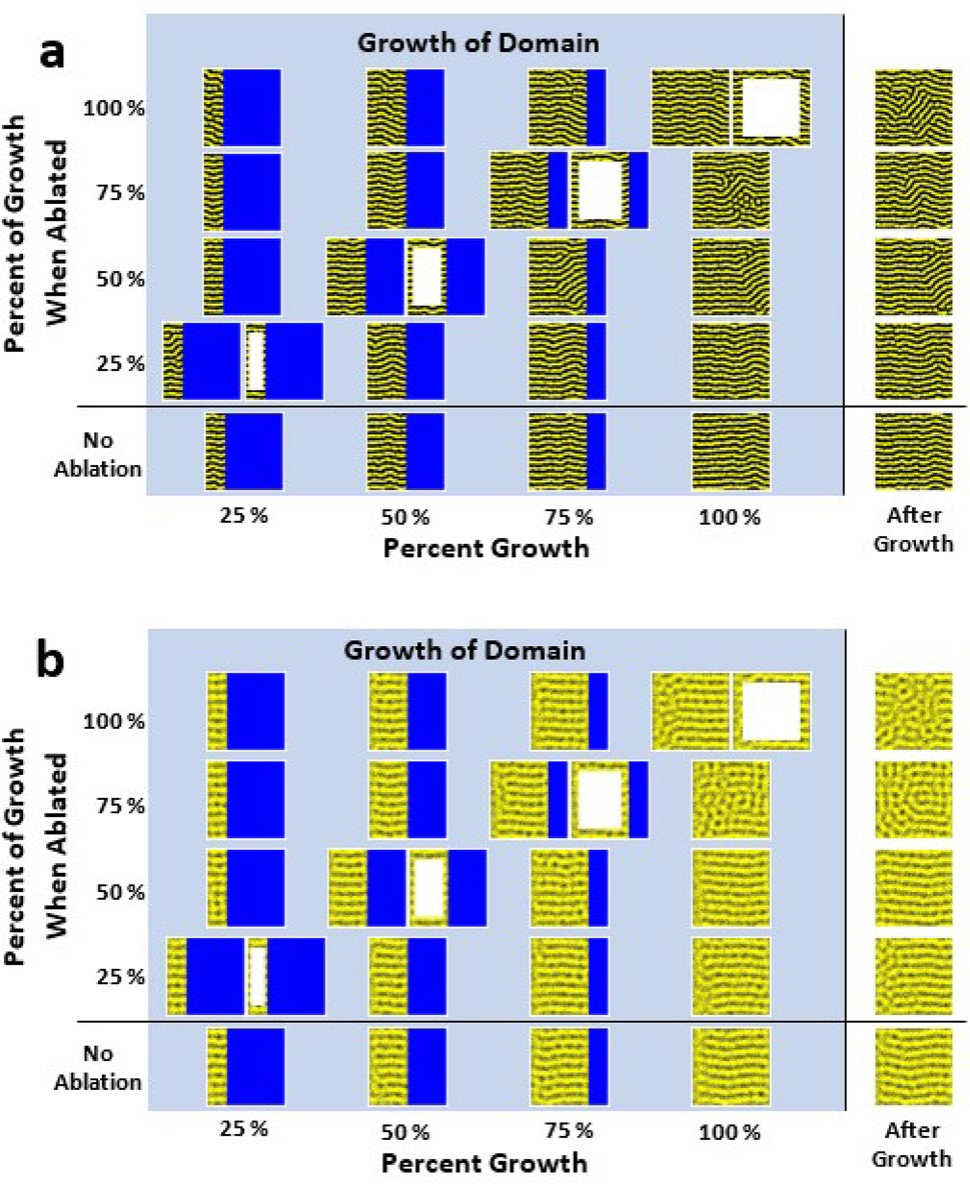
Ablation of Turing patterns at different percentages of growth. Simulations with the same conditions (one per row) were ablated at 25%, 50%, 75%, 100% of their total growth. The middle 75% of the domain was ablated (right side of each dual image—left side is simulation directly before ablation). Once the growth was complete, the simulations continued to run for $\approx 17\%$ of the time of growth to observe the stability of the final pattern (far right of each simulation). (**a**) Growth simulations of the Promotion model with ablation. The domain grows from a $300 \times 1$ cell lattice to a $300 \times 300$ cell lattice. The conditions of each simulation (rows) are: $h = 14, b_X = 1, s = 1, l_X = 2.5, b_M = d_M = d_X = 0$. (**b**) Growth simulations of the Survival model with ablation. The domain grows from a $400 \times 1$ cell lattice to a $400 \times 400$ cell lattice. The conditions of each simulation (rows) are: $h = 30, b_X = 1, s = 1, b_M = 7, d_M = 9, d_X =d_{M X} = 0$.

Laser ablation of zebrafish chromatophores has often been used to study the cellular mechanisms that form their skin patterns^14,15,21,22,28,29^. To simulate this behavior, we “ablated” the central lattice sites of growing pattern simulations at different percentages of growth (Fig. 6). To ablate the pattern, we replaced the middle 75% of the rows and columns in the simulation with empty cells (white) in the stochastic Monte Carlo simulations. The simulations then continued to grow until they reached their final size, and then the simulation was continued on a static domain to see if the resulting patterns were spatially stable (Fig. 6, “After Growth” column). A simulation with no ablation is shown in the bottom row of Fig. 6a and b for comparison. Animations of the the simulations ablated at 50% and 100% for both the Promotion model (Fig. 6a) and the Survival model (Fig. 6b) are included in the Supplementary Information.

Simulations of the Promotion and Survival models (Fig. 6a and b, respectively) with ablation show very similar behavior. If ablation occurs once the growth is complete (top row in Fig. 6a,b), the patterns will reform in a random orientation where the ablation occurs (rightmost column in Fig. 6a,b). However, if ablation occurs early during growth (for example, 25% or 50% of growth completed), the pattern recovers to an orientation perpendicular to the growing boundary, as if the pattern had not been ablated at all (shown for comparison in bottom rows of Fig. 6a,b). These behaviors are qualitatively very similar to what occurs for both fully-developed adult^14,15^ and developing^44^ zebrafish when their patterns are ablated.

## Discussion

We have proposed the “Survival model,” a simplified reaction scheme that can generate Turing patterns on a lattice in the absence of cellular movement. Instead, Gierer and Meinhardt’s SRALRI conditions are met *via* short-range competition between the two chromatophores (leading to self-activation) and having xanthophores enhance the survival of melanophores at longer distances (Fig. 1). This model is inspired by multiple experimental studies on zebrafish pattern formation^19,21,22,45^. In particular, the “Survival” feedback corresponds to a Delta/Notch signalling pathway found in adult zebrafish: melanophores extend a projection towards xanthophores, which then carries a signal essential to melanophore survival^15,22,23^. These projections reach a maximum size of half the stripe width. The Survival model is particularly applicable to the patterns on the tail and anal fins of the zebrafish. Unlike the body, where iridophores are necessary for pattern formation^18,43^, the patterns formed on the tail and anal fins are produced without other chromatophores present^44,45^. In addition, melanophore movement is heavily restricted on the zebrafish fins, justifying the approximation of immobile cells^44,46^.

The Survival model in this paper incorporates many of the same interactions as the previously-published Promotion model^31^, as shown in Fig. 1. The only significant difference is the nature of the long-range interaction. When comparing the Monte Carlo simulations of the Survival lattice on a static domain (Figs. 3a, 4) to those of the Promotion model in Reference^31^, one can see that the melanophore stripes/spots are much less dense in the Survival model. This may indicate that a “Survival” interaction constitutes a weaker form of feedback than a “Promotion” interaction. Yet, even this weaker long-range interaction is sufficient to induce Turing patterns over a wide variety of conditions (Figs. 2, 3). It is also worth noting that the more diffuse melanophore stripes resemble the melanophore stripes found on actual zebrafish^15^ more closely than the more homogeneous stripes seen in the Promotion model simulations.

In addition to studying a new type of long-range interaction in the Survival model, we have also investigated the impact of domain growth, a process that has not previously been considered in either the Survival or the Promotion model. Our stochastic simulations of the two models on growing domains indicate that growth has a major impact on pattern development and orientation. The simulations in Figs. 5 and 6 show that growth can orient patterns perpendicular to the growing boundary. Even when a defect in the pattern occurs, such as ablation, if the system is still growing, the pattern will spontaneously reorient itself. These are particularly interesting results as the lattice nodes themselves are not actually moving - only new nodes are added. Yet, similar behavior is shown in other, more traditional reaction-diffusion systems on growing domains in experimental^36,37,47^, agent-based numerical^29,30^, and analytical^9,32,33,35^ studies. These results may not demonstrate unequivocally that domain growth alone is responsible for the parallel stripe orientation on zebrafish tail and anal fins, but they do indicate that domain growth may play a significant role in ensuring that zebrafish patterns form along the growth axis in a reproducible manner^21,22,30,48^.

While the Survival and Promotion models behave qualitatively similarly on a growing domain (Fig. 5), simulations of the Survival model require a much large *h* value (the long-range interaction distance) to orient perpendicular to the growing boundary. In most zebrafish, the stripes are approximately 10-20 cells wide. Yet, in the Survival model with growth, the perpendicular orientation only occurs with a stripe width of at least 30 cells ($h \ge 30$) for the reaction parameters used here. This suggests that during zebrafish growth, other types of cellular interactions are likely to be involved in the initial pattern formation. One possible such interaction could be caused by the airinemes described in Eom et al.^21^. Airinemes are protrusions extending from xanthophores located inside melanophore stripes to nearby melanophores. They cause melanophores to consolidate into stripes during earlier stages of development, but then retract as zebrafish reach maturity^21^.

Our studies of the Survival model, its behavior on a growing domain and during ablation have yielded several interesting results. We have shown that zebrafish pattern formation, particularly on the fins, may arise from a Turing bifurcation with a new form of the long-range interaction - which was inspired by experimental studies^22^. The patterns that result from simulations of this model are consistent with the “Differential Growth” picture previously proposed by Bullara and De Decker^31^ in that Turing-type patterns can arise without morphogen movement, even when the Survival feedback is a weaker form of feedback than the Promotion feedback. In addition, we showed that domain growth can have a significant impact on the pattern orientation for both the Promotion and Survival models. The growing domain can orient the resulting patterns (albeit at different long-range interaction distances depending on the model), just as it can for traditional reaction-diffusion systems with morphogen diffusion^35–37,49,50^. In the future, it should be possible to update our model to account for additional intercellular interactions in order to provide a more complete understanding of the morphogenesis occurring on the skin of the zebrafish. It may also be of interest to examine pattern-forming behavior in other natural systems, such as the positional information-guiding cytonemes in *drosophila*^51^ or the reaction-diffusion-advection models of synaptogenesis in *C. elegans*^52^, using the modeling techniques employed in this work.

## Methods

### Theoretical approach for the lattice-based model

To model the zebrafish skin, we use a lattice of size *N* where each node $i = 1,2,...,N$ can be occupied by either a a xanthophore ($X_i$) or a melanophore ($M_i$) or remain empty ($S_i$). We define the “state” of the system by specifying the occupancy of each node (*X*, *M*, or *S*). Thus, there are $3^N$ possible states of the system. Transitions between states are determined in a probabilistic manner based on the rate constants of the various interactions.

We use two approaches to simulate the system and show that patterns can develop. The first method is stochastic simulation using a Monte Carlo algorithm which is described in detail in a later subsection of the Methods. Each simulation is one realization of the system evolving in time via a Markovian process - where at most one event can occur per unit time. Examples of non-growing simulations of this type are shown in Figs. 3a and 4. The second method of simulation is deterministic mean-field simulation, whose results are shown in Figs. 2 and 3b,c in the main text. Instead of simulating one instance of the system’s time evolution, the mean field equations (Eqs. (1)–(2) in the main text) describe the average behavior of an ensemble of realizations of the system. A full description of the equations and methods used for the deterministic mean-field simulations is given later in the Methods. In addition, a set of continuous mean-field equations (Eqs. (3)–(4) in the main text) can be derived from the mean field equations using a second-order Taylor series expansion. These are used to show that a Turing-type mechanism guides the pattern formation in the Survival model.

### Turing analysis

To determine whether the continuous mean field equation system is capable of undergoing a Turing-type bifurcation, we perform a linear stability analysis on a simplified version of Eqs. (3) and (4). Specifically, we are looking for a homogeneous steady state that is stable without the spatially dependent terms, but becomes unstable when the cross diffusion-like terms are added. We define a general reaction-diffusion system as

7 $$\begin{aligned} \frac{\partial \varvec{c}}{\partial t} = \mathscr {R}(\varvec{c}) + \mathscr {D} \nabla ^2 \varvec{c} \end{aligned}$$

where $\varvec{c}$ is a vector of morphogen concentrations, $\mathscr {R}(\varvec{c})$ are the reaction terms, and $\mathscr {D}$ is a matrix of the diffusion coefficients. We calculate the steady states (in the absence of diffusion) by solving

$$\begin{aligned} \mathscr {R}(\varvec{c_0}) = \varvec{0} \end{aligned}$$

and denote the Jacobian $\mathscr {J}$ of the reaction vector function $\mathscr {R}$ at the point $\varvec{c_0}$ as

$$\begin{aligned} \mathscr {J} = \frac{d \mathscr {R}(\varvec{c})}{d \varvec{c}} \Bigr |_{\varvec{c} = \varvec{c_0}} \end{aligned}$$

A Turing instability occurs when the system is in a stable steady state at $\varvec{c_0}$, and then is destabilized by a spatial perturbation of nonzero wavenumber *k*. The steady state $\varvec{c_0}$ is stable with respect to spatially homogeneous perturbations when the real parts of the eigenvalues of the Jacobian matrix $\mathscr {J}$ are all negative, which in a two-variable systems occurs when

8 $$\begin{aligned} Tr(\mathscr {J}) < 0 \text { and } Det(\mathscr {J}) > 0 \end{aligned}$$

To examine the spatial instability at wavenumber *k*, we calculate the linearized matrix of the system (Eq. 7), $\mathscr {L}$, in the form

9 $$\begin{aligned} \mathscr {L} = \mathscr {J} - k^2 \mathscr {D} \end{aligned}$$

If $\mathscr {L}$ has a positive eigenvalue (indicating an instability) for a finite, positive wavenumber *k*, then a Turing bifurcation has occurred, which produces spatially periodic patterns which are stationary in time. To show this, we calculate the characteristic equation of the eigenvalue $\omega$, which in a two-variable system takes the form:

10 $$\begin{aligned} \omega ^2 - Tr(\mathscr {L}) \omega + Det(\mathscr {L}) = 0 \end{aligned}$$

For the system to be unstable and stationary in time, the eigenvalue $\omega$ must be greater than zero and have no imaginary component ($\omega >0$ and $\text {Im}(\omega ) = 0$). For the system to be periodic in space, the wavenumber $k \ne 0$. Thus, if we can show that the eigenvalue $\omega$ is positive for a positive finite value of *k*, then a Turing instability exists for the system.

We can also analytically solve for the Turing bifurcation point; that is, the value at which a perturbation with critical wavenumber $k_T$ becomes unstable. To solve for the bifurcation point, we solve the systems of equations:

11 $$\begin{aligned} Det(\mathscr {L}) = 0 \quad \text { and } \quad \frac{{\partial } Det(\mathscr {L})}{{\partial } k^2} = 0 \end{aligned}$$

for the critical wavenumber $k_T$ and the critical value of a bifurcation parameter (for the Survival model, we will solve for the critical long-range interaction distance, $h_T$). In the following subsections, we show that this analysis holds for a simplified version of the Survival model.

#### Linear stability analysis of nonuniform steady state

The continuous mean field equations (Eqs. (3)–(4)) are simplified using the assumptions given in the main text. In addition, we assume $a = 1$, which establishes the space scale. The resulting simplified PDE system is:

12 $$\begin{aligned}&\frac{\partial x}{\partial t} = b_X (1 - x - m) - s m x - \frac{1}{2} s x \nabla ^2 m \end{aligned}$$

13 $$\begin{aligned}&\frac{\partial m}{\partial t} = b_M (1 - x - m) - s m x - d_M m (1 - x) - \left( \frac{1}{2} s m - \frac{h^2}{2} d_M m \right) \nabla ^2 x \end{aligned}$$

The steady states of the system (Eqs. (12) and (13)) in the absence of the cross-diffusion terms are given by Eqs. (5) and (6) in the main text. No Turing bifurcation can occur at the homogeneous steady state (Eq. (5)), and thus it will not be analyzed further.

To determine the stability of the second steady state (Eq. (6)), we first calculate the Jacobian matrix of the reaction functions to determine how the functions react to small perturbations. The Jacobian is:

14 $$\begin{aligned} \mathscr {J} = \begin{pmatrix} -b_X - s m &{} -b_X - s x \\ -b_M + d_M m - s m &{} -b_M - d_M + d_M x - s x \end{pmatrix} \end{aligned}$$

The Jacobian yields a trace of

15 $$\begin{aligned} Tr(\mathscr {J}) = -b_M - b_X - d_M (1-x) - s (m + x) \end{aligned}$$

Since by definition $x \le 1$ and all rate constants, *x*, and *m* are greater than zero, every term in the trace is negative. When the determinant of the Jacobian is calculated ($Det(\mathscr {J})$) and the steady state values of $x = x_2$ and $m = m_2$ are substituted, the resulting equation is:

16 $$\begin{aligned} Det(\mathscr {J}) = (b_M - b_X) s \end{aligned}$$

This meets the stability requirements shown in Eq. (8) when $b_M > b_X$. Thus, in the absence of the diffusion-like terms, the steady state $(x_2, m_2)$ is stable per the conditions in Eq. (8).

Notice that if we substitute the values of *x* and *m* for the first steady states (5) into the condition for the trace (15) and the determinant (16) of the Jacobian we obtain

17 $$\begin{aligned} Tr(\mathscr {J}) = -b_M - b_X - s < 0 \quad \text { and } \quad Det(\mathscr {J}) = - (b_M - b_X) s \end{aligned}$$

which predicts that the uniform steady state becomes a saddle point (and therefore unstable) as $b_M > b_X$. In other words, for $b_M = b_X$ we have a *transcritical bifurcation* through which the two steady states of the systems exchange stability.

#### Turing bifurcation

To determine whether steady state $(x_2,\, m_2)$ is stable with regard to spatial perturbations, we first construct the “diffusion” matrix $\mathscr {D}$ as

$$\begin{aligned} \mathscr {D} = \begin{pmatrix} 0 &{} -\frac{s}{2} x \\ \frac{h^2}{2} d_M m - \frac{s}{2} m &{} 0 \end{pmatrix} \end{aligned}$$

From here, we can construct the linearized matrix $\mathscr {L}$ as per Eq. (9):

18 $$\begin{aligned} \mathscr {L} = \begin{pmatrix} -b_X - s m &{} -b_X - s x + k^2 \frac{s}{2} x \\ -b_M + d_M m - s m - k^2 \left( \frac{h^2}{2} d_M m - \frac{s}{2} m \right) &{} -b_M - d_M + d_M x - s x \end{pmatrix} \end{aligned}$$

We can then solve for the bifurcation parameter $h_T$ and critical wavenumber $k_T$ using the conditions in Eq. (11). A plot of twice the critical long-range interaction distance ($h_T$) is shown in cyan in Fig. 2. The critical wavenumber can also be converted into the critical wavelength $\lambda _T$ using the relationship

$$\begin{aligned} \lambda _T = \frac{2 \pi }{k_{T}} \end{aligned}$$

The blue curve plotted in Fig. 2 is $\lambda _T / 2$. Thus, for every value of $h > h_T$ the second steady state is unstable to linear spatial perturbations. It will form spatial patterns; however, the patterns can sometimes have very small amplitudes and thus are only visible under certain parameters (see Figs. 2a,b and 3b,c).

### Deterministic mean field simulations

We simulate the deterministic behaviors of the system for a static domain by numerically solving Eqs. (1) and (2) on a $n = 50$ one-dimensional lattice in Fig. 2. We perform the numerical simulations in MATLAB using the solver ode23s. The state of each node is given by the relative occupancy (ranging from 0 to 1) of each chromatophore. Normalized concentrations of xanthophores are shown in Fig. 2a,c. Simulations where the normalized concentrations were scaled between their maximum and minimum values are shown in 2b. The same methods were used to simulate the corresponding two-dimensional mean field equations on a 50 x 50 lattice in Fig. 3b,c.

### Stochastic Monte Carlo simulations

The actual simulations (described below) were performed using a custom-developed C++ package. Then, the .csv files that were exported during the simulations were converted to image files using the Python package zebrafish_plot. This package also produces the animations of growing simulations, examples of which are found in the Supplementary Information. Descriptions of the C++ and Python package algorithms are below, and a link to the code is given in Code Availability section.

#### Base algorithm with survival model

The simulation algorithm for the Survival model on a static domain is similar to the algorithm used in Bullara et al. for the Promotion model^31^. A brief description of the algorithm on a static (non-growing) domain is:

1. A rectangular lattice with periodic boundary conditions is generated with a predefined number of rows and columns. Each lattice node has four first neighbors and can be occupied by either an empty space (*S*, 0 in the exported .csv files), a xanthophore (*X*, 1 in the exported .csv files), or a melanophore (*M*, 2 in the exported .csv files). The lattice can be initialized as empty (all nodes are *S*), a random distribution of chromatophores and empty sites (random assortment of *X*, *M*, and *S*), or with all xanthophores or melanophores. In all of the simulations shown in this article, the lattice starts as empty.
2. Probabilities for each reaction shown in Fig. 1 for the Survival model are calculated as the rate constant divided by the sum of all of the rate constants.
3. A predefined number of iterations of the Monte Carlo algorithm is chosen to ensure that a stable pattern will form. For the static simulations in Figs. 3a and 4 in the main text, the number of iterations is set to $10^9$. For each iteration, one lattice node (say position *k*) is selected at random, and one of the reaction process shown in Fig. 1 in the main text is selected at random with the appropriate probability. If the process selected involves another lattice node (short-range or long-range interactions), a nearest neighbor (short-range interaction) or node a distance *h* away (long-range interaction) is chosen at random to determine whether the process condition is met. If the conditions for the process are met, the lattice is updated.
4. Comma-separated values files (.csv) of the two-dimensional lattice state are exported after a specific number of iterations, as defined in the code.
5. After all iterations are complete, the final lattice is exported along with a .csv file containing all of the relevant parameters used in the simulation.

#### Implementing domain growth and ablation

The above algorithm was modified to include domain growth. Instead of defining the size of the domain and the number of iterations of the Monte Carlo algorithm steps, we choose the initial size of the domain, the number of iterations before growth, and the number of growth events. When the simulation reaches the desired number of iterations, new lattice nodes are added to one side, and the nearest neighbors are updated with the new nodes. The added nodes can either be identical to an adjacent column or all empty. For all of the growth shown in Figs. 5 and 6 in the main text, an empty column was added at each growth event, which occurred every $10^7$ iterations. There are functions in the Code Availability section that allow for trapezoidal growth, where both rows and columns are added. In addition, the user can specify the number of iterations before growth starts or after it is complete.

In a similar manner, ablation is performed by setting a code variable to specify when the user wants to ablate the system. When the simulation reaches the point of ablation, it converts a percentage of the middle rows and middle columns to empty sites (*S*). For the simulations shown in Fig. 6, the middle 75% of rows and the middle 75% of columns were ablated.

#### Converting output to image files and animations

Once a simulation is complete, the exported .csv files were converted to the individual .png files shown in Figs. 3a, 4, 5, and 6 as well as Supplemental Figs. S1 and S2 in Python. For each imported .csv file, an array of the largest array size was created and was colored according to the numeric value in the .csv file. For xanthophores (value of 1 in the .csv file), the image array was colored yellow, and for melanophores (value of 2 in the .csv file), the image array was colored black. Empty sites were left as white, and if the image array was larger than the current .csv file array (because growth had not been completed yet) the remainder of the image array was colored blue. Each of the resulting image arrays was exported to a subfolder within the folder containing the original .csv files. Then, the image files were knitted together into an animation, which was saved into the same subfolder.

## Supplementary information

Supplementary Information 1.

Supplementary Information 2.

Supplementary Information 3.

Supplementary Information 4.

Supplementary Information 5.

Supplementary Information 6.

Supplementary Information 7.

## Data Availability

The code developed for this study is available in the EpsteinLab Github. The Survival-MC and Zebrafish-Plot repositories specifically are used. Link: https://github.com/EpsteinLab.
