# Supplementary material for: Influence of survival, promotion, and growth on pattern formation in zebrafish skin
Source: Sci Rep. 2021 May 10;11:9864. doi: 10.1038/s41598-021-89116-4 (PMC8110552; doi:10.1038/s41598-021-89116-4)
Supplement: Supplementary file 1 — Supplementary Information 1. [file 41598_2021_89116_MOESM1_ESM.pdf]

Supplementary Information: Influence of survival, promotion, and growth on pattern formation in zebrafish skin

Christopher Konow, Ziyao Li, Samantha Shepherd, Domenico Bullara, and Irving R. Epstein

Supplementary Figures

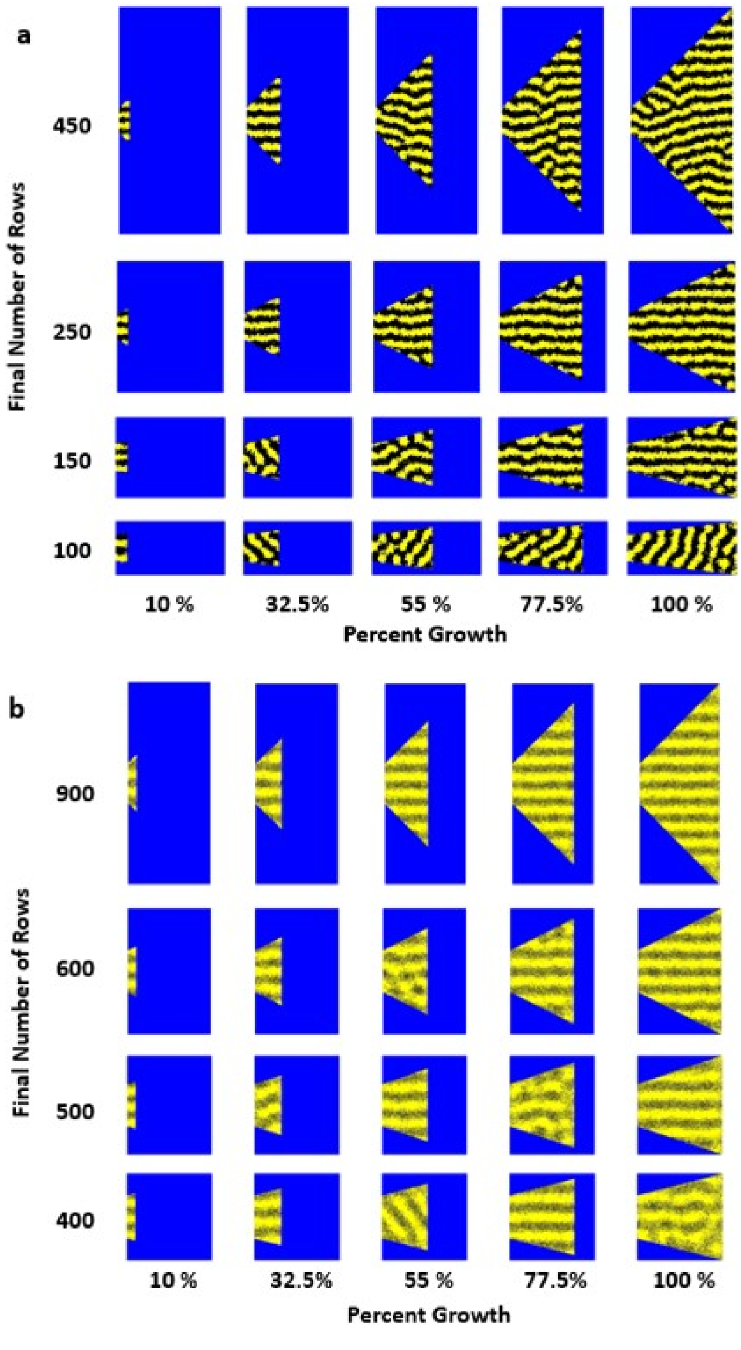

Figure S1: Turing pattern development during domain growth on a trapezoidal geometry. In each simulation (rows), images are shown of the developing pattern at 10%, 32.5%, 55%, 77.5%, and 100% of growth. All simulations are performed with periodic boundary conditions for the same simulation length. a) Stochastic Monte Carlo simulations of the Promotion model on a trapezoidal growing domain. Each simulation begins as a 50 x 1 lattice, and grows to be a size of 200 columns, with the number of rows given by the value on the left of the simulation. Each simulation is performed with $h = 14$, $b_X = 1$, $s = 1$, $l_X = 2.5$, $b_M = d_X = d_M = 0$. b) Stochastic Monte Carlo simulations of the Survival model on a trapezoidal growing domain. Each simulation begins as a 200 x 1 lattice, and grows to be a size of 400 columns, with the number of rows given by the value on the left of the simulation. Each simulation is performed with $h = 50$, $b_X = 1$, $s = 1$, $b_M = 7$, $d_M = 9$, $d_X = d_{MX} = 0$.

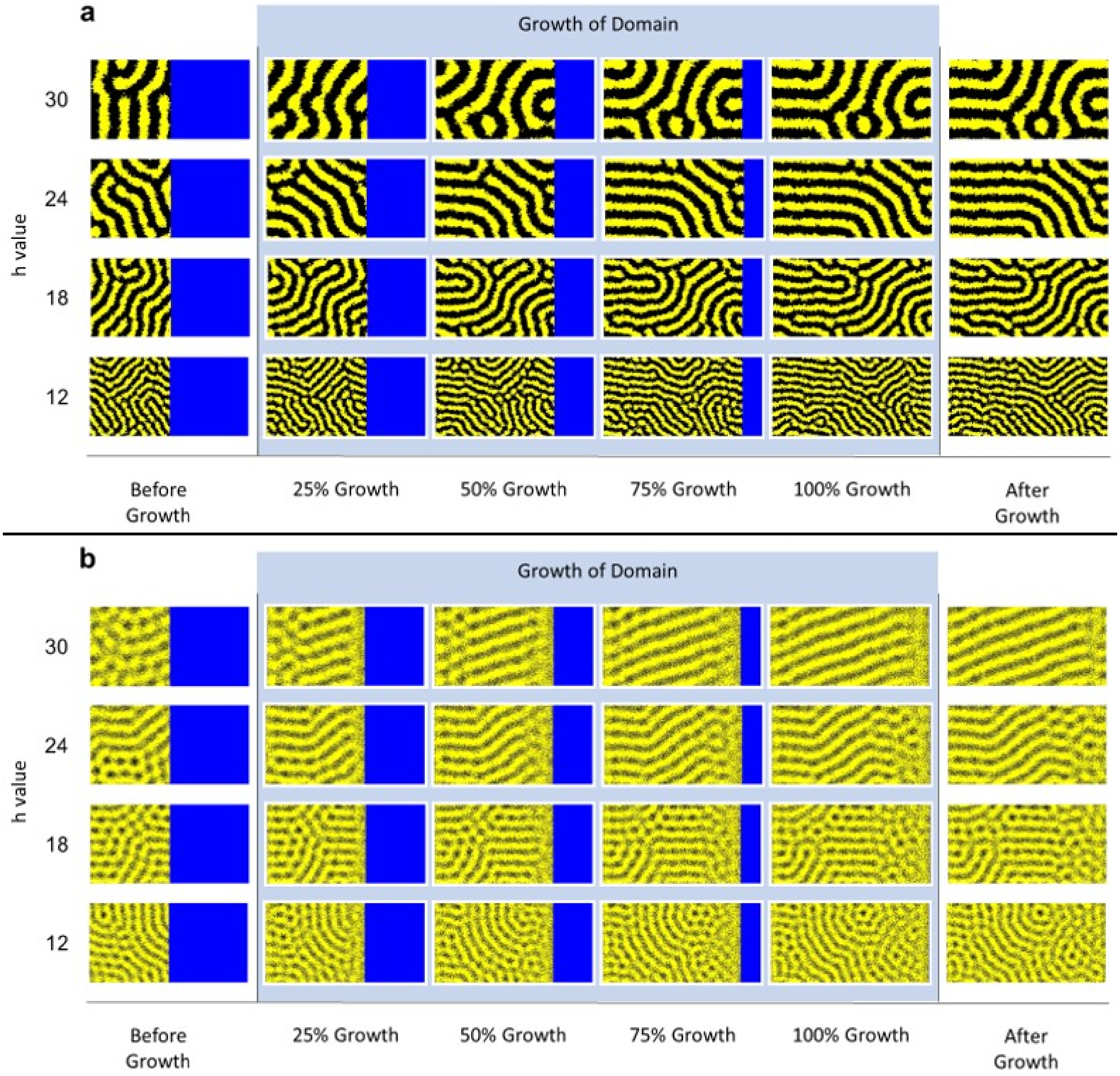

Figure S2: Development of Turing patterns with growth from an originally static, randomly patterned domain. The pattern developed for 25% of the total time (Before Growth), grew to twice its size (Growth of Domain), and then continued forming on the final domain for an additional 25% of the total time (After Growth). A) Simulations of the Promotion model. Each simulation started on a static 200 x 200 lattice and then grew to a final domain size of 200 x 400. The simulations were performed with rate constants $b_X = s_X = s_M = 1$, $l_X = 2.5$, and $b_M = d_X = d_M = 0$. B) Simulations of the Survival model. Each simulation started on a static 400 x 400 lattice and then grew to a final domain size of 400 x 800. The simulations were performed with rate constants $b_X = s_X = s_M = 1$, $b_M = 7$, $d_M = 9$, and $d_X = d_{MX} = 0$.

Supplementary Videos

Growth animations

- Supplementary Video S1: Promotion model Turing pattern formation on a growing domain with $h = 10$.
- Supplementary Video S2: Promotion model Turing pattern formation on a growing domain with $h = 16$.
- Supplementary Video S3: Survival model Turing pattern formation on a growing domain with $h = 20$.
- Supplementary Video S4: Survival model Turing pattern formation on a growing domain with $h = 50$.

Ablation animations

- Supplementary Video S5: Ablation of Turing pattern formed via the Promotion model at 50% of the growth completed.
- Supplementary Video S6: Ablation of Turing pattern formed via the Survival model at 50% of the growth completed.
